# Nkx2.2 and Nkx2.9 Are the Key Regulators to Determine Cell Fate of Branchial and Visceral Motor Neurons in Caudal Hindbrain

**DOI:** 10.1371/journal.pone.0124408

**Published:** 2015-04-28

**Authors:** Wassan Jarrar, Jose M. Dias, Johan Ericson, Hans-Henning Arnold, Andreas Holz

**Affiliations:** 1 Cell and Molecular Biology, Zoological Institute, University of Braunschweig, Braunschweig, Germany; 2 Department of Cell and Molecular Biology, Karolinska Institute, Stockholm, Sweden; Texas A&M University, UNITED STATES

## Abstract

Cranial motor nerves in vertebrates are comprised of the three principal subtypes of branchial, visceral, and somatic motor neurons, which develop in typical patterns along the anteroposterior and dorsoventral axes of hindbrain. Here we demonstrate that the formation of branchial and visceral motor neurons critically depends on the transcription factors Nkx2.2 and Nkx2.9, which together determine the cell fate of neuronal progenitor cells. Disruption of both genes in mouse embryos results in complete loss of the vagal and spinal accessory motor nerves, and partial loss of the facial and glossopharyngeal motor nerves, while the purely somatic hypoglossal and abducens motor nerves are not diminished. Cell lineage analysis in a genetically marked mouse line reveals that alterations of cranial nerves in Nkx2.2; Nkx2.9 double-deficient mouse embryos result from changes of cell fate in neuronal progenitor cells. As a consequence progenitors of branchiovisceral motor neurons in the ventral p3 domain of hindbrain are transformed to somatic motor neurons, which use ventral exit points to send axon trajectories to their targets. Cell fate transformation is limited to the caudal hindbrain, as the trigeminal nerve is not affected in double-mutant embryos suggesting that Nkx2.2 and Nkx2.9 proteins play no role in the development of branchiovisceral motor neurons in hindbrain rostral to rhombomere 4.

## Introduction

In vertebrates the cranial motor nerves control the muscles on which eye, head and neck movements, swallowing, sound formation and facial expressions depend. Cell somata of cranial motor neurons are partitioned into distinct nuclei residing in well-defined areas of the brainstem including midbrain and hindbrain. The vast majority of motor neurons localizes to the hindbrain, which during embryonic development becomes segmented along the rostrocaudal axis. These functionally and molecularly distinct units are referred to as rhombomeres which obtain their individual identity by the expression of a specific combination of Hox genes in the particular segment [1]. Hox gene patterns are controlled, at least in part, by the diffusible signals FGF8 and retinoic acid present in rostral and caudal sections of hindbrain, respectively [2, 3]. The precise molecular definition of the anteroposterior (a-p) axis in hindbrain is crucial, because path finding of individual cranial nerves to muscles of the eye, the tongue, lower jaw, neck, or parasympathetic ganglia is governed by their a-p rhombomeric position.

Both rostrocaudal and dorsoventral patterning play essential roles in the development of hindbrain. Primarily based on experiments in spinal cord it has been proposed that sonic hedgehog (SHH) protein supplied by notochord and floor plate forms a ventral-to-dorsal concentration gradient within the spinal cord, and most likely also in hindbrain, which leads to dose-dependent differentiation of various types of neurons [4, 5]. Indeed, development of cranial motor neurons in hindbrain strictly depends on the presence of the signaling molecule SHH [6]. According to the patterning model in spinal cord graded SHH signaling would also govern the expression of homeodomain proteins in distinct domains along the dorsoventral axis in hindbrain [7]. Neuronal progenitor cells within the basal plate (ventral) are destined to differentiate into three cardinal subtypes of cranial motor neurons: branchiomotor neurons (bMN) that innervate branchial arch-derived muscles, visceral motor neurons (vMN) that project onto parasympathetic ganglia, and somatic motor neurons (sMN) that control somite-derived striated muscles [7]. Significantly, sMNs that constitute the abducens and hypoglossal nerves are restricted exclusively to rhombomeres 5 and 7, while vMNs of the facial, glossopharyngeal, and vagal motor nerves as well as bMNs that contribute to the trigeminal, facial, glossopharyngeal, vagal, and accessory motor nerves are generated in specific segments along the rostrocaudal axis with the exception of rhombomere 1. These observations indicate the influence of the axial position on the development and specification of motor neuron subtypes.

The most ventral region that harbors neuronal progenitor cells dorsal to the floor plate is referred to as p3 domain. It gives rise to branchial and visceral motor neurons in hindbrain, while the next dorsally adjacent pMN domain generates somatic motor neurons [8, 9]. Cell bodies of sMNs remain in the ventral position and their axons leave the CNS ventrally, whereas somata of bMNs and vMNs migrate dorsally toward the alar plate and their axons project to dorso-lateral exit points from which they navigate to their targets in the periphery. The specificity of these axonal projections is probably determined as part of the neuronal differentiation program directed by rostrocaudal and dorsoventral patterning cues.

We and others have previously shown by loss-of-function mutations in mouse that specification of progenitor cells in the p3 domain of spinal cord and their subsequent differentiation to V3 interneurons is dependent on overlapping functions of the transcription factors Nkx2.2 and Nkx2.9 [8, 10, 11]. Both proteins are very similar in sequence and presumably structure, and are co-expressed in the p3 domain along the entire rostrocaudal extension of the CNS [8, 12]. It therefore seems reasonable to assume that Nkx2.2 and Nkx2.9 proteins might also collaborate as key regulators in hindbrain to generate bMNs and vMNs in the p3 domain. Individual mutations of either gene did not result in a general loss of these motor neurons, although disruption of Nkx2.9 caused a moderate defect of the spinal accessory nerve that exclusively contains bMNs [10, 13]. On the other hand, ablation of the Nkx2.2 gene had no effect on the formation of motor neurons but caused extensive but not complete loss of serotonergic interneurons that normally develop at later embryonic stages from progenitor cells in the p3 region of all rhombomeres except r4 [8, 14]. Functional redundancy of Nkx2.2 and Nkx2.9 proteins might provide a plausible explanation for the lack of a severe motor neuron phenotype in hindbrains of individual single gene knockout mutants, although functional overlap between both genes has not been demonstrated experimentally.

In this study we provide evidence for essential and redundant roles of the Nkx2.2 and Nkx2.9 transcription factors in the formation of cranial motor nerves of the caudal hindbrain and the specification of branchial and visceral motor neurons. Significantly, the double-null mutation exerts no effect on the development of branchial motor neurons anterior to rhombomere 4 suggesting that additional factor(s) for specification of bMNs must exist in the rostral hindbrain. We also demonstrate that the neuronal progenitor cell lineage in the p3 domain is transformed to the sMN fate in the absence of both Nkx2.2 and Nkx2.9 factors, even in rhombomeres 4 and 6, which are normally devoid of somatic motor neurons. These supernumerary sMNs contribute to enlarged nuclei of somatic cranial motor nerves indicating that the entire differentiation program of these extra sMNs has changed including their migration and axonal projections.

## Material and Methods

### Mouse strains

Nkx2.2; Nkx2.9 double-knockout mice were obtained by crossing Nkx2.2- [15] and Nkx2.9-deficient mice [10]. Genotyping was performed as described previously [11]. Generation of the Nkx2.2^Cre^ knockin mouse line will be published elsewhere (Jarrar et al, in press, doi:10.1016/j.diff.2015.03.001). In this mouse the entire Nkx2.2 open reading frame was replaced by the coding sequence of Cre recombinase. It was genotyped by PCR using the primer combination Nkx2.2 sense (5’- TGC TTT CCG AGA AGA GAG AGG CA), Nkx2.2 antisense (5’- TTG TCG CTG CTG TCG TAG AAA GG), and iCre antisense (5’- ACA GTC AGC AGG TTG GAG ACT TTC CTC). A 450 bp and 283 bp fragment represent the wild type and mutant allele, respectively. Cre-mediated recombination was monitored on Rosa-mT/mG [16] and Rosa26-R/lacZ [17] reporter mouse strains. The morning of vaginal plug detection was counted as E0.5.

All procedures concerning animals were approved by the animal welfare representative of the TU Braunschweig and the Niedersächsisches Landesamt für Verbraucherschutz und Lebensmittelsicherheit (Oldenburg, Germany)(Permit Number: 33.14-42502-04-10/0203).

### Immunohistochemistry

Antigens were identified on cryosections of samples from embryos that have been fixed in 4%- paraformaldehyde as described previously [11]. Primary antibodies and sera used in this study were anti-Phox2b (1:500; H-20, Santa Cruz Biotechnology), anti-Phox2b (1:500)[18], anti-Hb9 (1:400; 81.5C10, DSHB), anti-neurofilament (1:800; 2H3, DSHB), anti-Nkx2.2 (1:1000; 74.5A5, DSHB), anti-Islet1 (1:600; 40.2D6, DSHB), anti-Islet1 (1:600; 39.4D5, DSHB), anti-Nkx6.1 (1:800; F55A10, DSHB), anti-active caspase 3 (1:300; ab2302, Abcam), anti-GFP (1:800; MAB3580, Millipore), anti-GFP (1:2000; A-11122, Molecular Probes), anti-Olig2 (1:400; 18953, IBL), and anti-ß-galactosidase (1:10000; #559761, Cappel). All secondary antibodies were purchased from Jackson Immunoresearch and added to sections for 2 hours at 1:300–1:600 dilutions in PBS. Sections were then counterstained with DAPI (Molecular Probes). Images were acquired on a Zeiss Axioplan 2 fluorescence microscope or on Olympus BX61W1 FluoView 1000 confocal laser scanning microscope.

For immunohistochemistry of whole-mount embryos, samples were fixed with methanol/DMSO (4:1) at 4°C overnight and bleached with methanol/DMSO/H_2_O_2_ (4:1:1). After rehydration embryos were incubated in the appropriate blocking solution containing 5% serum/0.1% Tween20/PBS. Primary antibodies were applied overnight at 4°C using the following dilutions: anti-neurofilament (1:800; 2H3, DSHB), anti-NCAM-L1 (1:500; C-20, Santa Cruz Biotechnology), anti-Hoxb1 (1:200; PRB-231P, Covance), and anti-GFP antibody (1:2000; A-11122, Molecular Probes). Following incubation embryos were washed thoroughly prior to adding the appropriate HRP-conjugated antibody (all Jackson Immunoresearch). The DAB/H_2_O_2_ staining reaction was performed and whole-mount images of embryos were documented with a digital color camera mounted on the Nikon stereomicroscope SMZ1000.

### *In situ* hybridization

Serial transverse cryostat sections of the brainstem were fixed with 4% paraformaldehyde/PBS and were hybridized at 68°C with a digoxigenin-labeled riboprobe for murine *peripherin* mRNA. Digoxigenin-labeled hybrids were identified by the AP-conjugated antibody (1:2000, Roche) as described previously [11]. Images were taken on a Leica DM-RBE microscope.

### Evaluation of data

For each experimental group at least 3 individual embryos with ≥3 sections were evaluated. Results are shown as means ± standard deviation. Cells were counted using Adobe Photoshop software. For statistical evaluations the Student’s t-test and one-way ANOVA analyses were performed by comparing wild type and mutant animals using GraphPad software.

## Results

### Both Nkx2.2 and Nkx2.9 transcription factors control the formation of cranial motor nerves in hindbrain

**Fig 1:**
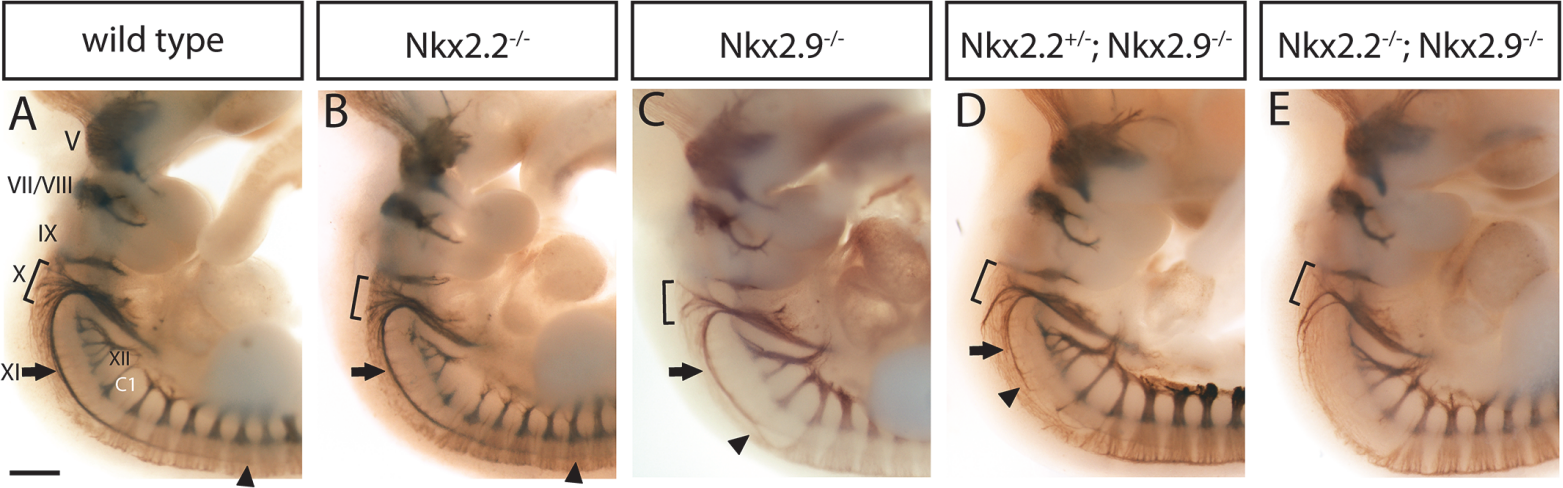
Immunostaining of cranial motor nerves in whole-mount mouse embryos: Effects on the spinal accessory and vagal nerve in mutant mouse embryos lacking Nkx2.2 and/or Nkx2.9 genes. Lateral view of brainstem including the anterior part of the developing spinal cord of wild type (A), Nkx2.2^-/-^ (B), Nkx2.9^-/-^ (C), Nkx2.2^+/-^; Nkx2.9^-/-^ (D), and Nkx2.2^-/-^; Nkx2.9^-/-^ (E) embryos at E10.5. Immunostaining of motor neurons was performed with NCAM-L1-specific antibody. The arrow indicates the accessory nerve (XI) with the arrowhead marking its maximal caudal extension. The rootlets of the vagal nerve (X) are indicated by the bracket. In wild type and Nkx2.2^-/-^ single knock-out embryos, the spinal accessory nerve extends from rhombomere 7 into the cervical spinal cord (see arrowheads) and the vagal nerve appears also normal. In the absence of Nkx2.9, however, the N. accessorius is shorter and thinner than usual (arrowhead in C) and the vagal nerve exhibits moderate defects. Nkx2.2 and Nkx2.9 compound and double-null mutant embryos show almost total loss of the accessory nerve with only few unorganized axon fascicles remaining and severe defects of the vagus nerve illustrated by the severe reduction of rootlets (D and E). Cranial nerves are referred to as trigeminal nerve (V), N. facialis/N. vestibulocochlearis (VII/VIII), N. glossopharyngeus (IX), N. vagus (X), N. accessorius (XI), and N. hypoglossus (XII). C1: first spinal nerve of the cervical spinal cord. Scale bar: 400 μm.

The combined contribution of the homeodomain transcription factors Nkx2.2 and Nkx2.9 to development of the hindbrain was determined on whole-mount preparations showing the cranial motor nerves of E10.5 wild-type, single mutant, and compound-mutant mouse embryos. The antibody directed against the neural cell adhesion molecule (NCAM-L1) reliably marks developing nerves. We found in embryos lacking either one or both transcription factors that most cranial nerves were present and appeared morphologically similar to those in wild type. These nerves included the trigeminal, facial, vestibulocochlear, glossopharyngeal, and hypoglossal nerves (Fig 1). In contrast, the spinal accessory nerve was almost completely missing in Nkx2.2/Nkx2.9 double-deficient embryos and the vagal nerve was substantially reduced in size with considerably fewer rootlets (Fig 1). This severe phenotype was consistently observed in double-null mutant embryos. On the other hand, in single-Nkx2.2^-/-^ knockout embryos the spinal accessory nerve and the vagal nerve were essentially normal (Fig 1A and 1B) but both nerves appeared slightly smaller in the Nkx2.9^-/-^ mutant (Fig 1C)[10]. Compound-mutant Nkx2.2^+/-^; Nkx2.9^-/-^ embryos containing only a single functional Nkx2.2 gene copy exhibited an intermediate phenotype with a considerably shorter and thinner spinal accessory nerve and a significantly diminished vagal nerve (Fig 1D). Comparable results were obtained in wild type and mutant embryos using the antibody 2H3 against a neurofilament protein (S1 Fig). This supports the idea that the corresponding motor neurons have been lost rather than just the expression of NCAM protein [19](see below). It seems unlikely that the defects observed for both cranial motor nerves at E10.5 were merely due to a developmental delay, since the same analysis performed on older double-null mutant embryos at E11.5 showed the identical nerve abnormalities (data not shown). Taken together these observations suggest that the transcription factors Nkx2.2 and Nkx2.9 play an essential and partly overlapping role in the formation of cranial motor nerves. This function becomes particularly obvious for the spinal accessory nerve and the N. vagus. Additional defects of other cranial nerves could not be ruled out at this point of our analysis, as changes on motor components might be obscured by sensory axons which are present in common fascicles and co-stain in the whole-mount analysis [20].

### The number of branchiovisceral motor neurons in hindbrain is drastically reduced in Nkx2.2 and Nkx2.9-double-deficient mouse embryos

The vagus and the spinal accessory nerve consist of branchiovisceral motor neurons (bvMN) and both nerves were severely affected in the Nkx2.2; Nkx2.9 double-deficient mouse mutant, whereas the hypoglossal nerve containing only somatic motor neurons (sMN) appeared essentially unaltered. We compared the relative numbers of both types of motor neurons in wild type and the various Nkx2.2; Nkx2.9 single and compound mutant mice. To identify postmitotic bvMNs the transcription factor Phox2b was utilized as marker on open-book preparations of hindbrains from E10.5 wild type and mutant embryos [21, 22]. Phox2b staining in the ventral midline was massively diminished in caudal hindbrain of Nkx2.2^-/-^; Nkx2.9^-/-^ double-knockout embryos compared to single mutants and wild type animals (S2 Fig). The reduction of Phox2b seemed to follow a posterior to anterior gradient with no or only little decrease in rhombomere 4 and rostral to it suggesting that the ventral expression domain of Phox2b in the anterior hindbrain was not or not entirely dependent on the Nkx2.2 and Nkx2.9 regulators. The population of Phox2b^+^ cells in the dorsal and lateral positions of the hindbrain representing primarily sensory neurons [23] was totally unaffected in all Nkx2 single and double mutant animals.

**Fig 2:**
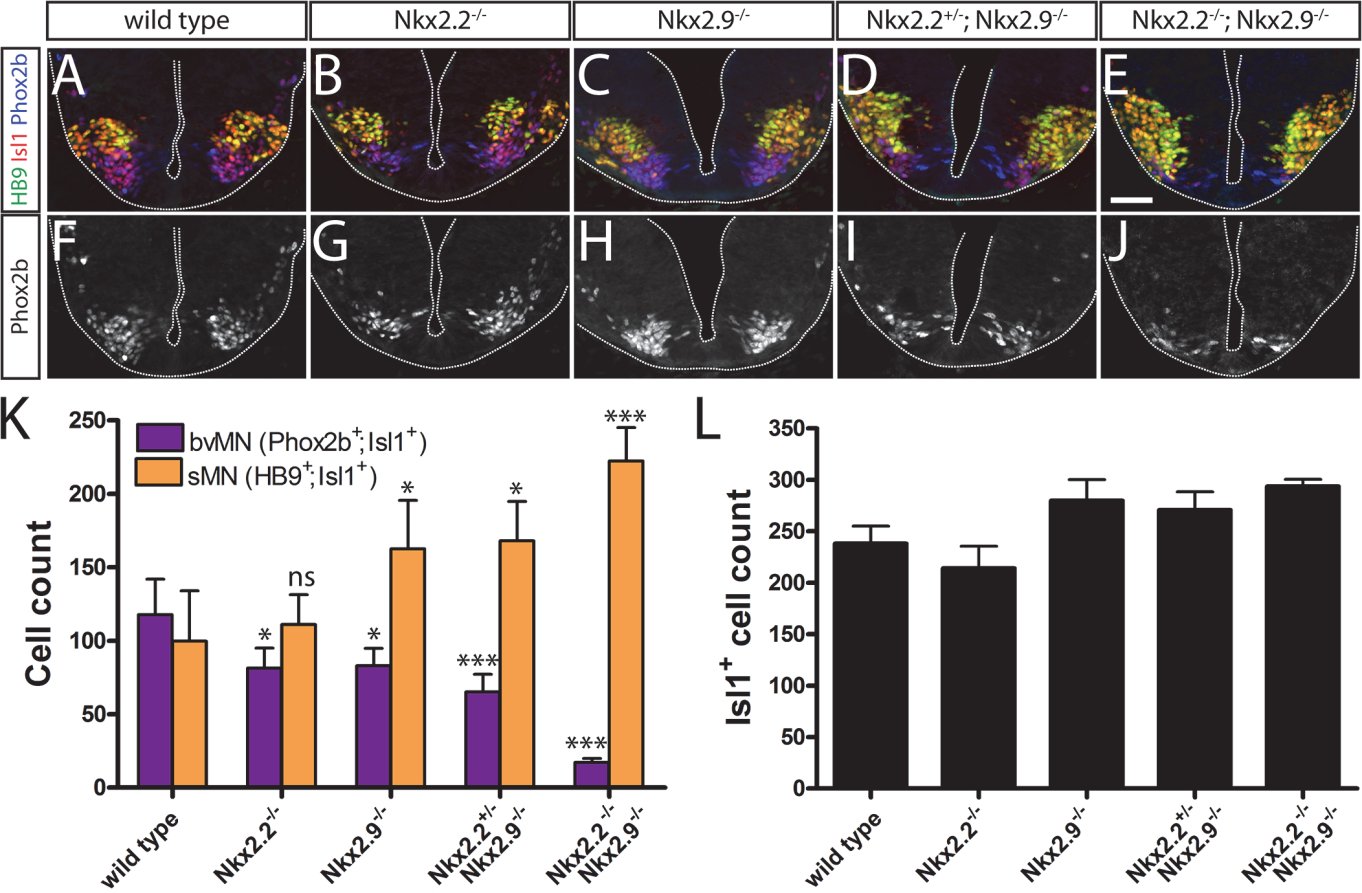
Reduction of branchiovisceral motor neurons in rhombomere 7 of the Nkx2.2; Nkx2.9 double-null mutant mouse embryo. Transversal sections through rhombomere 7 of wild type (A and F, n = 4), Nkx2.2^-/-^ (B and G, n = 3), Nkx2.9^-/-^ (C and H, n = 4), Nkx2.2^+/-^; Nkx2.9^-/-^ (D and I, n = 4), and Nkx2.2^-/-^; Nkx2.9^-/-^ (E and J, n = 5) embryos at E10.5. Branchiovisceral motor neurons were stained by immunohistochemistry for Phox2b^+^ in blue and Islet-1^+^ in red. Somatic motor neurons were stained for HB9^+^ in green and Islet-1^+^ in red. The decrease of Phox2b^+^ cell nuclei in mutant tissue is also illustrated for better recognition in black and white (F-J). Note that the decrease of bvMNs in mutant tissue appears to be counterbalanced by the inverse expansion of somatic motor neurons. The significance of this observation was determined by the one-way ANOVA statistical test (K). The total number of postmitotic Islet-1^+^ motor neurons was similar in wild type and the various Nkx2 mutant mice with small, statistically insignificant differences (L). ns: not significant, *: p < 0.05, ***: p < 0.001. Scale bar in E represents 50 μm and applies to all figure panels.

Loss of bvMNs in hindbrain of Nkx2.2- and Nkx2.9-deficient embryos was investigated in more detail by triple-immunofluorescence staining of post-mitotic motor neurons on transverse sections at the level of rhombomere 7 (Fig 2). In hindbrain of E10.5 wild type embryos comparable numbers of HB9^+^/Isl1^+^ somatic and Phox2b^+^/Isl1^+^ branchiovisceral motor neurons were detected in their characteristic positions, while in NKx2.2; Nkx2.9 double-null embryos most bvMNs were lost and apparently replaced by sMNs (Fig 2K). Moderately but significantly reduced numbers of bvMNs and slightly increased amounts of sMNs were also observed in single Nkx2.2 and Nkx2.9 mutants as well as in the Nkx2.2^+/-^; Nkx2.9^-/-^ compound mutant. Interestingly, the total number of motor neurons, identified by the pan-MN marker Islet1 (Isl1) [24], was not significantly different in wild type and mutant mouse embryos suggesting that bvMNs in mutant brain were probably not lost by cell death but rather transformed into the fate of sMN cells (Fig 2L). This interpretation seems supported by the observation that the ventral region in hindbrain which usually contains Phox2b^+^/Isl1^+^ bvMNs in wild type mice was primarily populated by HB9+/Isl1+ sMNs in double null-mutant animals (Fig 2A and 2E). We have no indication that the few Phox2b^+^/Isl1^-^ cells that were consistently observed in the ventricular zone of wild type and double null- mutant embryos will ever develop into bvMNs (Fig 2E and 2J).

**Fig 3:**
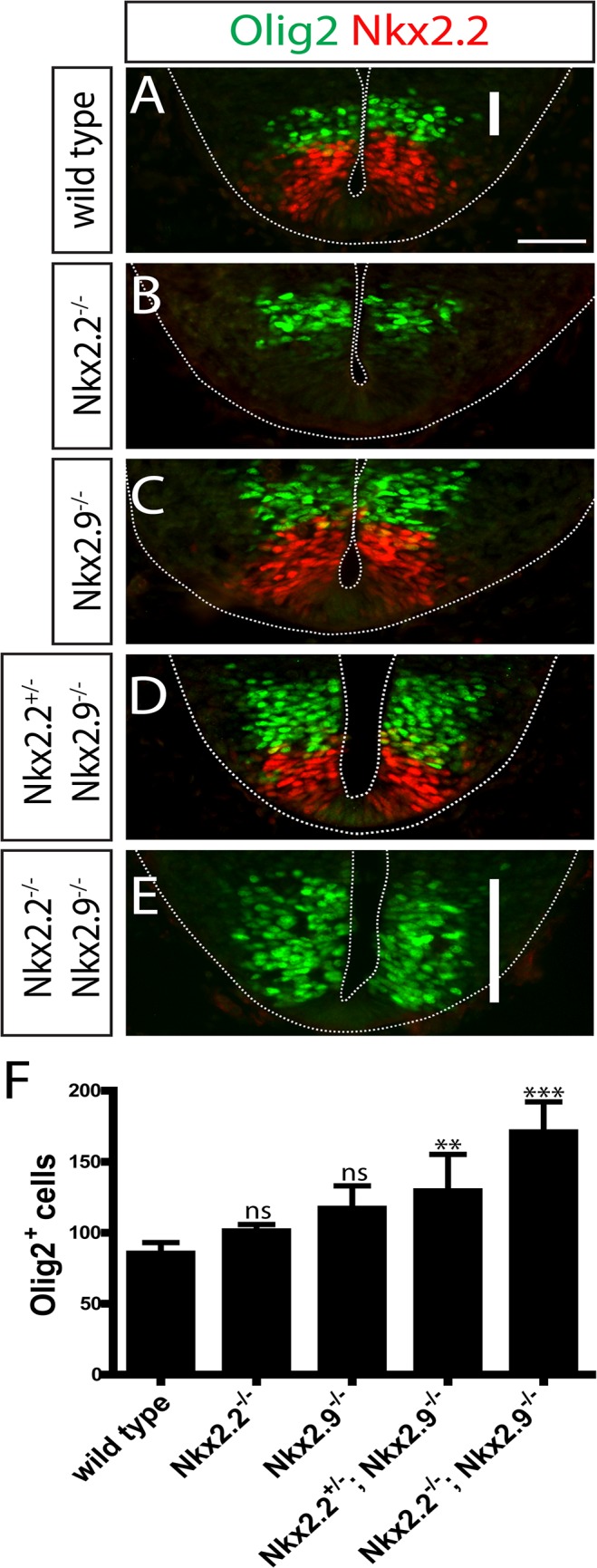
The p3 domain in hindbrain of Nkx2.2; Nkx2.9 double-deficient mutant embryos harbors progenitor cells for sMNs and lacks the normal population of bvMN progenitors. Immunofluorescence staining of Nkx2.2- (red) and Olig2- (green) expressing cells on transversal sections through rhombomere 7 in E10.5 mouse embryos (A-E). The ventricular and pial surfaces are indicated by dotted lines. The p3 domain characterized by bvMN progenitor cells (red) in wild type (A; n = 6), single Nkx2.2 (B; n = 4), and Nkx2.9 (C; n = 3) mutant embryos is moderately reduced in Nkx2.2^+/-^; Nkx2.9^-/-^ compound mutants (D; n = 6). These bvMNs progenitors are totally absent in Nkx2.2^-/-^; Nkx2.9^-/-^ double-null mutant embryos (E; n = 5) and have been replaced by Olig2^+^ SMN progenitor cells (indicated by white vertical bars in A and E). Olig2^+^ progenitors were counted on the sections through neural tubes and the columns represent the numbers in various mutants and control animals (F). Statistical significance was evaluated by one-way ANOVA analysis (ns: not significant, **: p < 0.01, ***: p < 0.001). Scale bar (white horizontal bar in A): 50 μm.

Next we examined bvMN progenitor cells in hindbrain (rhombomere 7) of E10.5 embryos. These progenitor cells are normally born in the Nkx2.2-positive p3 domain [8] immediately ventral to the Olig2-positive domain of sMN progenitors (Fig 3A)[25]. Using Nkx2.2 and Olig2 as molecular markers sMN progenitor cells were detected in double-null mutant embryos not only in their normal area dorsal to the p3 domain (Fig 3A–3C) but also within the p3 domain extending ventrally to the floor plate (normally occupied by Nkx2.2+ cells)(Fig 3E). This phenotype was not observed in embryos carrying single Nkx2 mutations suggesting that both Nkx2.2 and Nkx2.9 transcription factors contribute and are individually sufficient to determine bvMN progenitor cells in the p3 domain (Fig 3B and 3C). Taken together these results provide compelling evidence that Nkx2.2 and Nkx2.9 transcription factors are necessary and sufficient to specify progenitor cells of bvMNs in hindbrain possibly by preventing the alternative cell fate of somatic MNs, similar to their roles in V3 interneurons in spinal cord [11].

### A subset of motor nuclei of individual cranial nerves is missing or reduced in hindbrain of Nkx2.2; Nkx2.9 double-deficient mutant embryos

**Fig 4:**
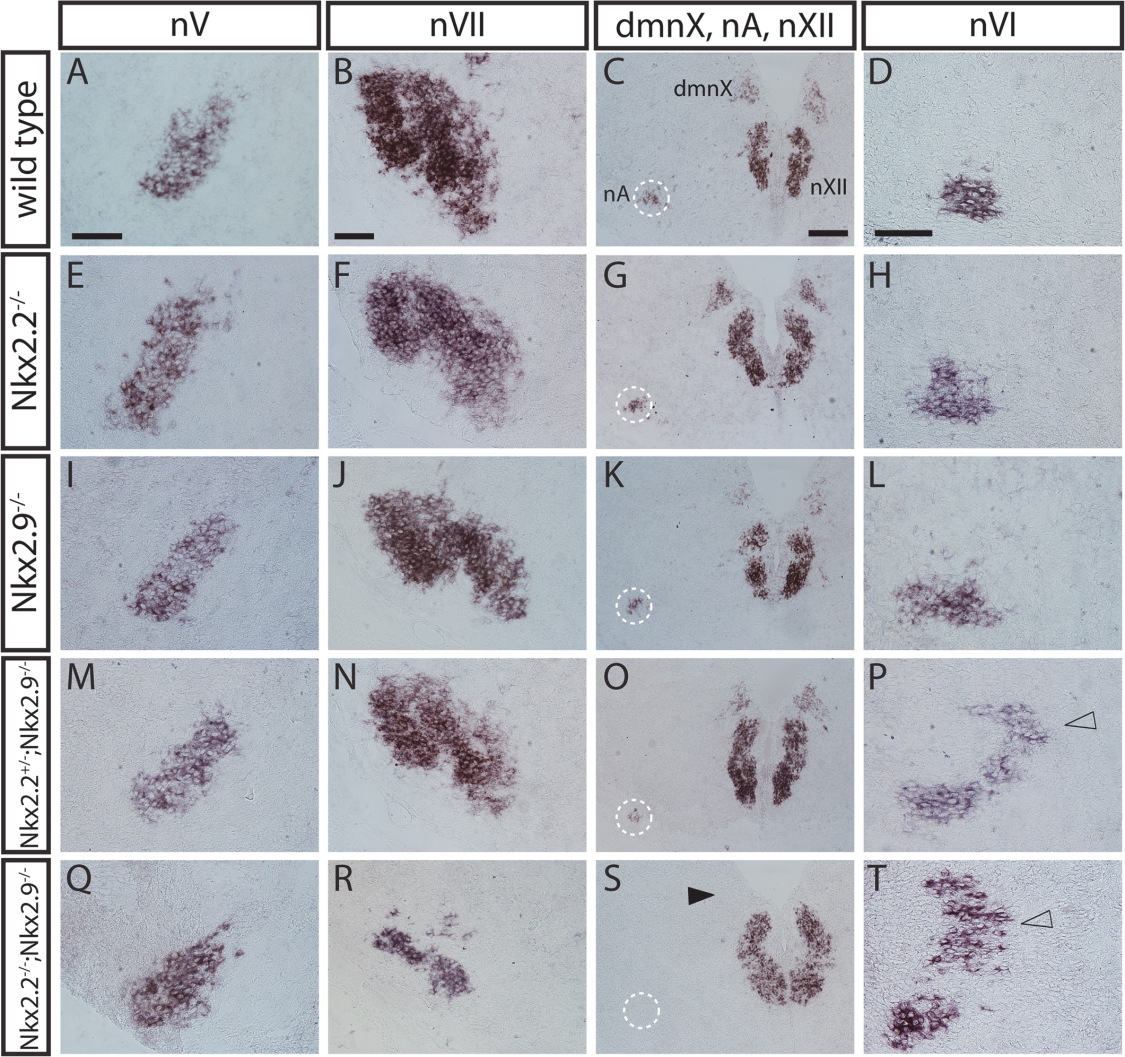
Changes in motor nuclei of cranial nerves in Nkx2.2^-/-^; Nkx2.9^-/-^ double-deficient mouse mutants. *In situ* hybridizations were performed with digoxigenin-labeled *peripherin* riboprobe on serial cross-sections of hindbrain from E15.5 wild type (A-D), single Nkx.2.2 (E-H) and Nkx2.9 (I-L), and double mutant (M-T) mouse embryos. Note the strong reduction of motor nuclei for the facial nerve (nVII) and the almost complete loss of nuclei for the vagal nerve (dmnX, filled arrowhead) as well as the nucleus ambiguus (dotted circle) in hindbrains of Nkx2.2^-/-^; Nkx2.9^-/-^ double-deficient embryos. In contrast, the branchiovisceral motor nucleus of the trigeminal nerve (nV) was basically unaltered and the somatic motor nuclei of the hypoglossal (nXII) and abducens (nVI) nerves appeared augmented in double mutant mice with additional ectopic *peripherin*-expressing cells next to the motor nucleus of the abducens nerve (open arrowhead in P and T). Scale bars correspond to 100 μm.

Given the considerable changes in the distribution of motor neuron subtypes and the axonal defects of the spinal accessory and the vagal nerves in hindbrain of Nkx2.2; Nkx2.9 double-deficient mutant embryos, we next studied the anatomical consequences of these altered pools of neurons on the formation of motor nuclei of individual cranial nerves. Transverse serial sections through hindbrains of E15.5 embryos were subjected to in situ hybridization using peripherin as a probe to identify motor nuclei. The nucleus ambiguus (nA) and the vagal motor nucleus dmnX were both entirely absent in double knock-out mice and markedly reduced in the single Nkx2.2 and Nkx2.9 mutants as well as in the Nkx2.2^-/+^; Nkx2.9^-/-^ compound mutant (Fig 4C, 4G, 4K, 4O and 4S). The facial nucleus was considerably smaller in double-null mutant embryos (Fig 4B and 4R), while the nucleus of the trigeminal nerve was apparently unaffected, although it contains branchial motor neurons (Fig 4A and 4Q). In contrast, motor nuclei of the abducens and hypoglossal nerves, which exclusively consist of sMNs, were both present and possibly enlarged in double-null and compound Nkx2.2^-/+^; Nkx2.9^-/-^ mutant embryos (Fig 4C, 4S and 4D, 4H, 4L, 4P, 4T). A considerable number of additional sMNs was detected near the nucleus abducens (Fig 4P and 4T). These results confirm and extend our previous observations on the relative reduction of bvMNs in favor of sMNs. The data also suggest that altered composition of neurons and the consequences for the development of cranial nerves occur to different extent depending on the rostrocaudal position in hindbrain of mutant mouse embryos.

### Nkx2.2 and Nkx2.9 transcription factors are required to determine bvMN progenitor cells in caudal but not in rostral hindbrain: Differential effects of the Nkx2.2; Nkx2.9 double mutation along the anteroposterior axis

**Fig 5:**
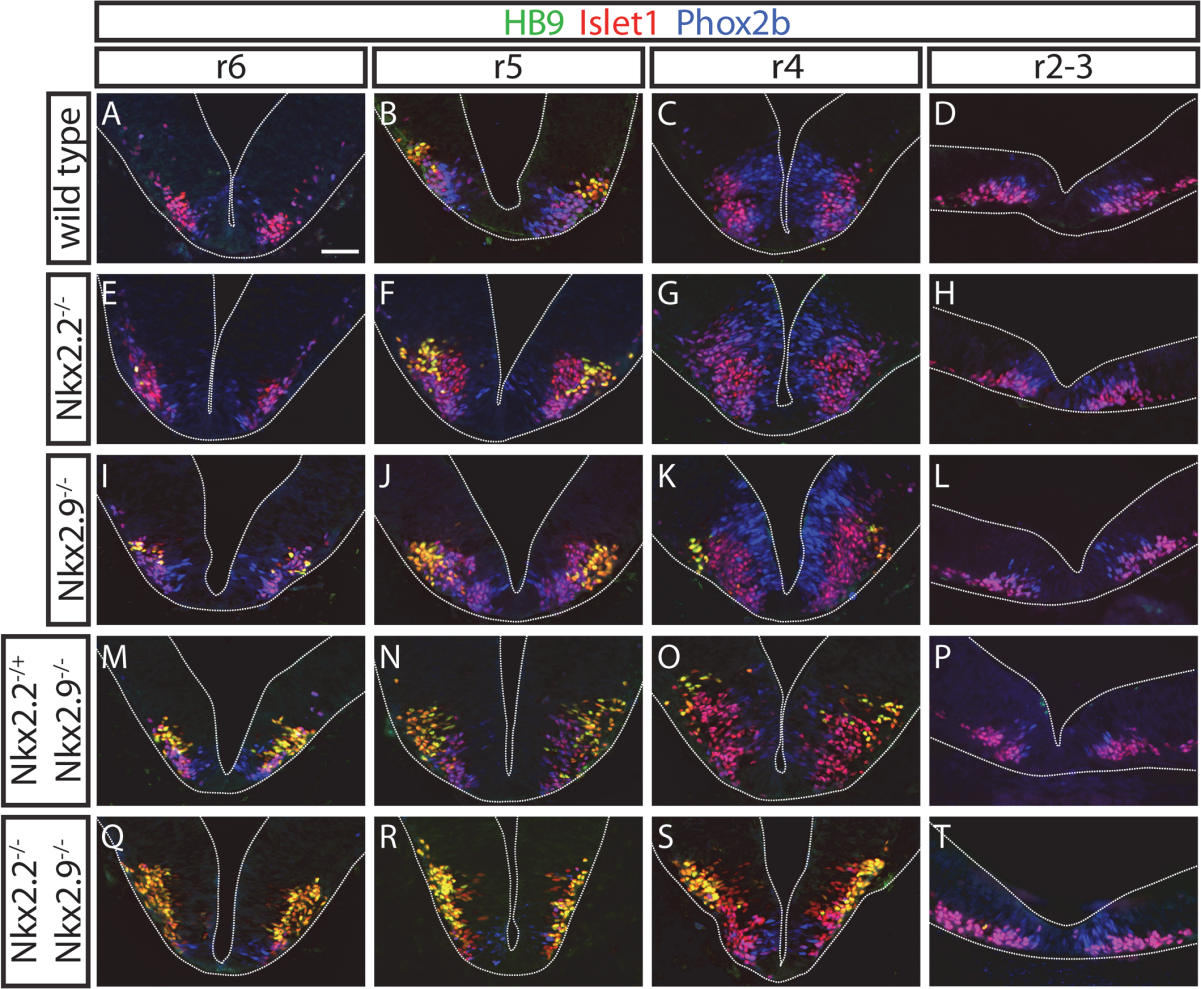
Additional and ectopic populations of somatic motor neurons develop in caudal but not in rostral hindbrain of Nkx2.2; Nkx2.9 deficient embryos. Triple immunofluorescence on transversal sections through hindbrains from wild type (A-D), Nkx2.2^-/-^ (E-H), Nkx2.9^-/-^ (I-L), Nkx2.2^+/-^; Nkx2.9^-/-^ (M-P), and Nkx2.2^-/-^; Nkx2.9^-/-^ (Q-T) E10.5 embryos reveals expression of HB9 (green)/Islet-1 (red) in somatic motor neurons, and Phox2b (blue)/Islet-1 (red) in branchial and visceral motor neurons. In wild type hindbrain somatic Hb9^+^/Islet-1^+^ double-positive motor neurons are only present in rhombomeres 5 (B) and 7 (see Fig 2), whereas hindbrains of Nkx2.2; Nkx2.9 double-knockout embryos contain considerable numbers of somatic motor neurons ectopically in rhombomeres 4 and 6 (Q-S). Similar albeit much weaker phenotypes have also been observed in single Nkx2.2 and Nkx2.9 knock-out and compound mutants (E-P). The surplus of sMNs is accounted for by significant loss of bvMNs (Q-S). The anterior rhombomeres 2 and 3 do not contain SMNs and failed to acquire ectopic sMNs even in the absence of both Nkx2.2 and Nkx2.9 transcription factors. Scale bar in A: 50 μm.

The observation that the motor nucleus of the trigeminal nerve appeared basically unaltered in Nkx2.2; Nkx2.9 double-null mutants prompted us to extend the analysis of Nkx2.2 and Nkx2.9 in determination and development of bvMN progenitor cells and their descendants along the anteroposterior axis of hindbrain. Transverse sections of rhombomeres 6 to 2 were triple-stained with fluorescent antibodies specific for HB9, Phox2b, and Isl1 (Fig 5). Similar to the observations on rhombomere 7 of Nkx2.2; Nkx2.9 double-deficient embryos the population of HB9^+^/Isl1^+^ sMNs was enlarged in rhombomere 5 and expanded ventrally into the p3 domain, while Phox2b^+^/Isl1^+^ bvMNs were almost entirely lost (Fig 5B and 5R). Surprisingly, a large number of HB9^+^/Isl1^+^ sMNs was also detected at the expense of bvMNs in rhombomeres 6 and 4 of double-null embryos (Fig 5A, 5Q and 5C, 5S), although these rhombomeres in wild type mice are usually devoid of sMNs [26]. The ectopic accumulation of sMNs occurred to a much smaller degree also in Nkx2.9 single and Nkx2.2^+/-^; Nkx2.9^-/-^ compound mutant embryos (Fig 5I, 5K, 5M and 5O). In contrast, the more rostral rhombomeres 2 and 3 in double-null mutant embryos did not exhibit major phenotypic changes neither with respect to the normal numbers of bvMNs nor to the presence of ectopic sMNs (Fig 4D, 4H, 4L, 4P and 4T). These results support the idea that the requirement of Nkx2.2 and Nkx2.9 transcription factors for the specification of bvMN progenitor cells in hindbrain varies along the anteroposterior axis. Anterior to rhombomere 4 neither Nkx2.2 nor Nkx2.9 protein appears to play a major role in the development of bvMNs, while both transcription factors are absolutely essential to maintain bvMN cell fate within and caudal to rhombomere 4.

### Depletion of branchial and visceral motor neurons in Nkx2.2; Nkx2.9 double-null embryos results from fate transformation of bvMN to sMN progenitor cells

The reduction of branchiovisceral motor neurons in hindbrain of Nkx2.2; Nkx2.9 double-deficient mouse embryos could be due to specific cell death or to transformation of cell fate. Since we found no evidence for enhanced apoptosis at this time of development (data not shown), we examined the possibility of cell transformation of bvMN progenitor cells and their acquisition of sMNs characteristics. To this end a Nkx2.2-Cre knock-in mouse was generated that allows to strictly follow the neuronal cell lineage that originates in the p3 domain (Jarrar et al, in press, doi:10.1016/j.diff.2015.03.001).

**Fig 6:**
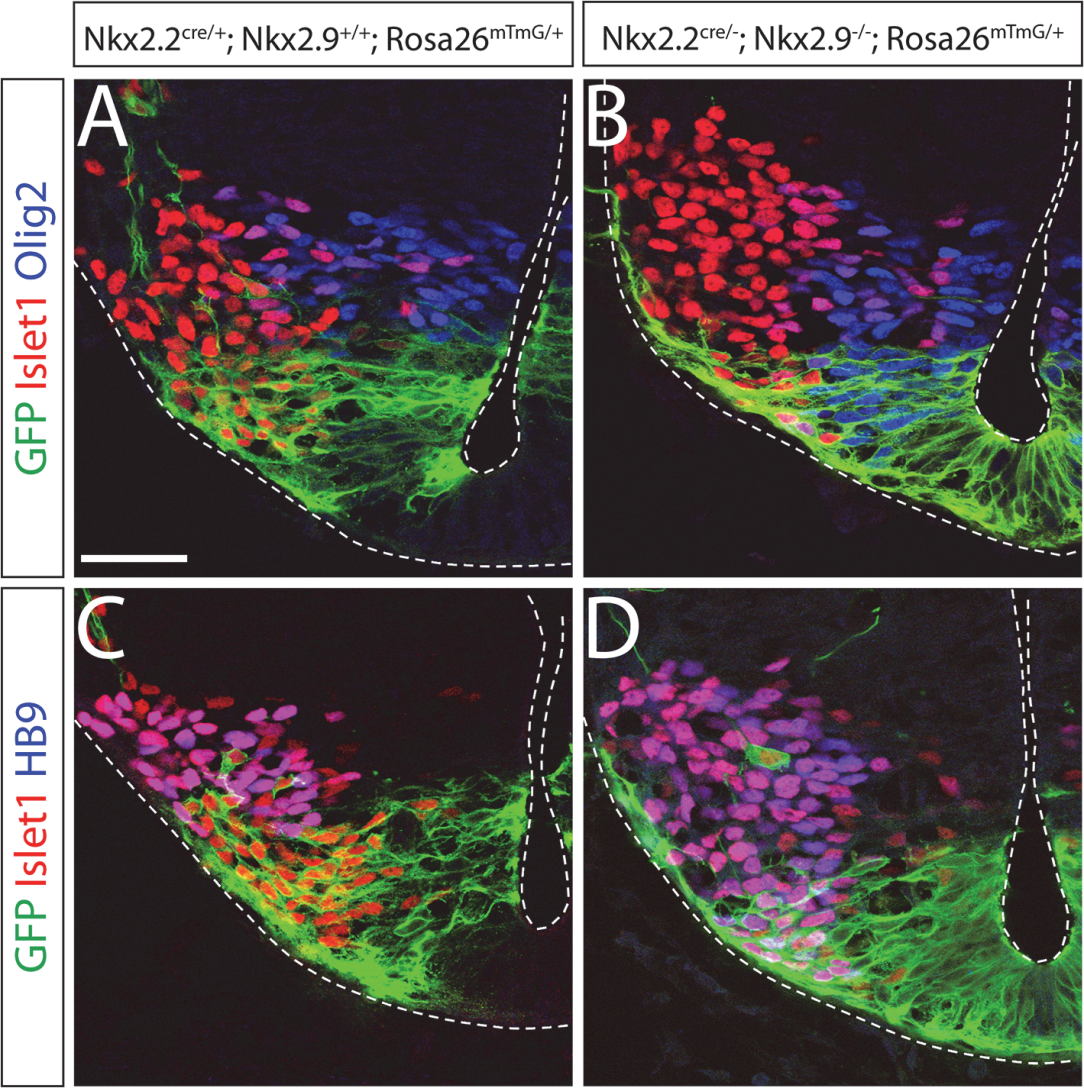
Transformation of motor neuron cell fate in hindbrain of Nkx2.2^-/-^; Nkx2.9^-/-^ double mutant embryos. Cells of the Nkx2.2-expressing bvMN lineage in the p3 domain were genetically labeled by membrane-targeted GFP (green) that is expressed persistently after Nkx2.2-Cre-mediated recombination in the Rosa-mT/mG reporter mouse. Confocal images of immunohistochemically stained sections from control (A, C) and Nkx2.2; Nkx2.9 double-deficient (B, D) mouse strains are shown. Note that p3 cells and their descendants in the presence of both Nkx2.2 and Nkx2.9 transcription factors (A, C) coexpress the cell lineage marker GFP together with the pan-motor neuron marker Islet1 (red) but fail to express the sMN markers Olig2 and HB9 (both blue). In contrast Nkx2.2^-/-^; Nkx2.9^-/-^ double mutant embryos exhibit lineage-marked GFP-positive neuronal progenitor cells that falsely also express the sMN progenitor-specific Olig2 (blue) and the postmitotic sMN marker HB9 in the p3 domain (B, D). Dotted lines delineate the ventricular and pial surfaces in the hindbrain sections. The scale bar in A represents 25 μm.

The Nkx2.2-Cre knock-in mouse was crossed with a tomato-GFP reporter mouse strain to visualize the Nkx2.2-expressing cells and their descendants via Cre-mediated accumulation of membrane-tagged GFP [16]. Transverse sections of rhombomere 7 demonstrated that lineage-marked GFP^+^ cells in control embryos were confined to the p3 domain, initially in the ventricular zone with bvMN progenitor cells and subsequently in the mantle zone with Isl1^+^ and HB9^+^ mature motor neurons (24; Fig 6A and 6C). These Isl1^+^ and GFP^+^ motor neurons in the appropriate p3 position also expressed the marker Phox2b confirming their identity as bvMNs (S3 Fig). Significantly, the population of GFP-marked cells in control embryos was dorsally sharply delimited by Olig2^+^ sMN progenitor cells and mature HB9^+^ sMNs that never expressed Nkx2.2 (Fig 6A and 6C). Some of the GFP-labeled bvMNs at E10.5 started already to migrate dorsally and sent out axonal projections at dorsolateral exit points [7](Fig 6A and 6B). In hindbrain of Nkx2.2; Nkx2.9 double-null embryos cells that contained membrane-associated GFP also expressed Olig2 in the progenitor cell compartment initially, and later co-expressed HB9 and Isl1 together with GFP in the more lateral region containing mature motor neurons (Fig 6B and 6D). These data clearly demonstrate that bvMNs were strictly derived from Nkx2.2-expressing progenitor cells in the p3 domain of the wild type mouse, while the identical p3 region of Nkx2.2; Nkx2.9-deficient mouse embryos generated cells that were derived from the same lineage-marked progenitors but now expressed typical markers of sMNs. Taken together these observations suggest that bvMN progenitor cells in Nkx2.2; Nkx2.9-deficient mouse embryos are subject to cell fate transformation resulting in sMNs that ectopically reside in the ventral domain typical of bvMNs.

### Axons of transformed sMNs leave the central nervous system correctly via ventral exit points

**Fig 7:**
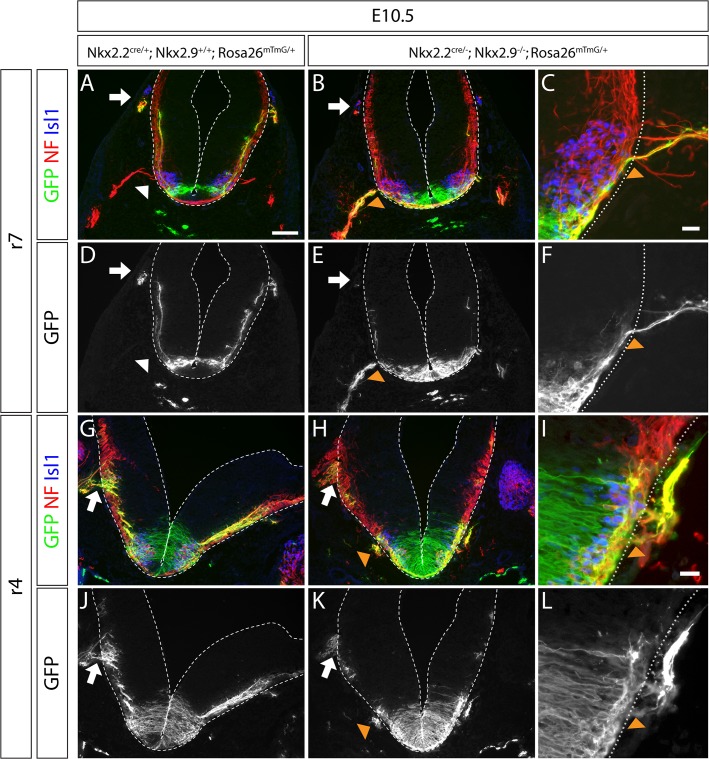
Ectopically generated somatic motor nerves in Nkx2.2; Nkx2.9 double-deficient mouse embryos project axons correctly via ventral exit points into the periphery. Hindbrain sections at levels of rhombomeres 7 (r7; A-F) and 4 (r4, G-L) from control (A, D, G, J) and Nkx2.2; Nkx2.9 double-deficient (B, C, E, F, H, I, K, L) mouse embryos (E10.5) were immunostained for GFP (green), neurofilament (NF, red) and Islet-1 (Isl, blue). Note that axons of the Nkx2.2-derived bvMN cell lineage leave the hindbrain exclusively through dorsolateral exit points, both in r4 and r7. These numerous axons in controls are labeled by membrane-targeted GFP (cell lineage marker: yellow in A, D, G, and J), while only few residual axons at best use this route in Nkx2.2; Nkx2.9 double-null mutants (arrows in B, E, H, and K). Axons of somatic motor nerves are identified by their expression of neurofilament (red label in A). Their projections leave the CNS exclusively via ventral exit points in r7, but not in r4 (white arrowheads in A and G). Axons transformed to the sMN subtype in Nkx2.2; Nkx2.9 double-null mutants mix with the original sMNs at preexisting ventral exit points of r7 (orange arrowheads in B, C, E, and F) and form additional ventral exit points in r4 (H, I, K and L). Scale bars: (A): 100 μm, (C) and (I): 20 μm.

Examination of the GFP-labeled p3 progenitor cell lineage in rhombomeres 4 and 7 of E10.5 wild type and mutant embryos provided further evidence for the complete phenotypic transformation of bvMN to sMN cell fate in the absence of Nkx2.2 and Nkx2.9 proteins. Axonal projections into the periphery of control and double-mutant embryos were routinely immuno-stained for neurofilament (red) and genetically labeled via membrane-tagged GFP (green) (Fig 7). In rhombomere 7 of control mice bvMN-derived axons displayed both markers (yellow) and left the brain exclusively at dorsolateral exit points, whereas sMN-derived axons did not express the lineage marker GFP and left the brain at ventral exit points (red) (Fig 7A and 7G). In Nkx2.2; Nkx2.9 double-deficient embryos the great majority of axons left the CNS at ventral exit points (Fig 7B and 7H). These axons were presumably projected from transformed sMNs, since they carried the membrane-bound cell lineage tag GFP (Fig 7C and 7F). Similar evidence for altered axonal routes was obtained in rhombomere 5 (data not shown). Remarkably, ventral exit points for sMN axons which are normally not present in rhombomeres 4 of wild-type mice (Fig 7H, 7I, 7K and 7L) were readily detected in double-null mutants based on the ectopic expression of neurofilaments (red) and the cell lineage marker GFP (green) (Fig 7H and 7I). Similar data were obtained in rhombomere 6 (data not shown). Taken together these observations suggest that novel sMN-specific ventral exit structures have been generated ectopically, possibly induced by axons emanating from sMNs that developed by cell fate transformation of bvMN progenitors. Notably, ventral exit points for axons were never observed in rostral rhombomeres 2 and 3 consistent with the lack of sMNs at this axial level (data not shown).

### Typical somatic motor nerves in the cervical region and caudal hindbrain of Nkx2.2; Nkx2.9 double-deficient mouse mutants are derived from the bvMN progenitor cell lineage

**Fig 8:**
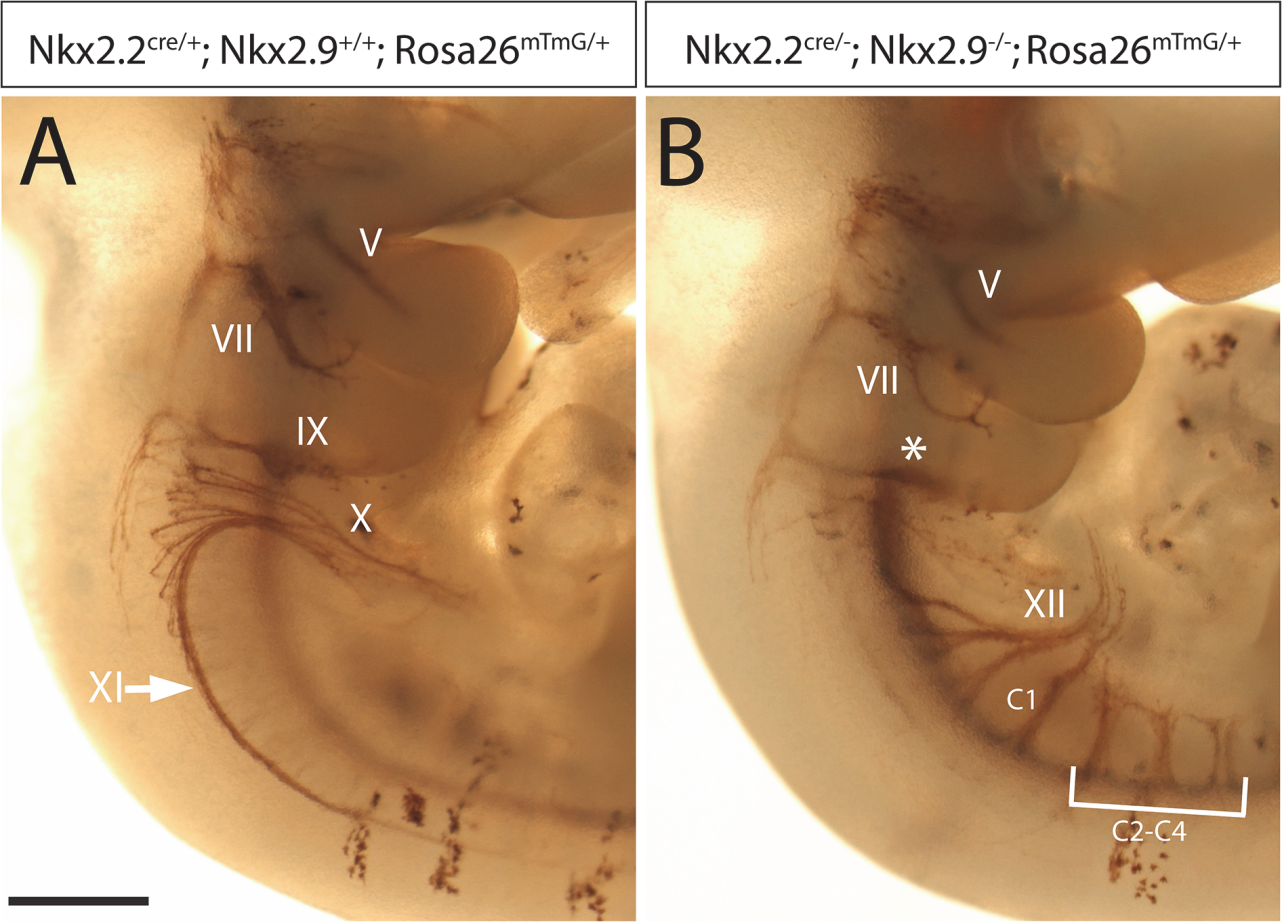
Typical somatic motor nerves are derived from progenitors of branchiovisceral motor neurons in Nkx2.2; Nkx2.9-deficient mutant embryos. Cre-mediated activation of membrane-targeted GFP is shown by immunostaining on whole- mounts of hindbrain and upper cervical spinal cord from control (A) and Nkx2.2; Nkx2.9 double-knockout (B) embryos. Axonal bundles derived from Nkx2.2-expressing progenitor cells (brown label) mark the branchial and visceral motor nerves in the E11.5 control mouse embryos (A). In the Nkx2.2; Nkx2.9-deficient mutant embryo the accessory (XI) and vagal (X) motor nerves are missing but axons of the hypoglossal nerve (XII) and ventral roots in cervical spinal cord are now labeled (B). The facial (VII) and glossopharyngeal (*) motor nerves are still present but markedly reduced (B). Note that the motor branch of the trigeminal nerve (V) in the mutant appears indistinguishable from control. Scale bar: 500 μm.

Whole-mount preparations of embryos were stained for the membrane-tagged GFP cell lineage marker to assess the origin of cranial motor nerves and somatic motor nerves in the spinal cord of Nkx2.2; Nkx2.9-deficient mouse mutants (Fig 8). Consistent with immunostaining of cranial nerves for NCAML1 and 2H3 neurofilament (Fig 1 and S1 Fig), axonal projections of the spinal accessory nerve and the vagal motor nerve were completely or nearly completely lost and those of the glossopharyngeal motor nerve were severely reduced in the double-knockout mutant embryos compared to controls (Fig 8A and 8B). In contrast the trigeminal and the facial nerves in double-null mutants appeared not or only partly affected, respectively. These observations were in perfect agreement with the cell lineage analysis of neuronal subtypes in the corresponding motor nuclei at E12.5 demonstrating that most if not all motor neurons in the trigeminal nucleus did not convert to sMN cell fate but retained the character of bvMNs (S4 Fig). By contrast, most bvMNs in the facial nucleus disappeared except for a small but significant number of residual bvMNs (S5 Fig). Interestingly, the axonal trajectories of the somatic hypoglossal nerve and ventral rootlets of the cervical spinal motor nerve in Nkx2.2; Nkx2.9-deficient animals also expressed the GFP cell lineage marker indicating that they were derived at least in part from Nkx2.2-activated cells. The axon bundles originating from transformed motor neurons were anatomically indistinguishable from those of the somatic motor nerves in wild type animals suggesting that sMNs that resulted from cell fate transformation in double-null mutant mice were fully capable of contributing to presumably functional somatic motor nerves in hindbrain and cranial spinal cord.

## Discussion

The molecular mechanisms underlying the specification of different neuronal subtypes that constitute the cranial motor nerves in vertebrates are not precisely defined. Particularly the individual roles of the transcription factors Nkx2.2 and Nkx2.9 in hindbrain development have remained unclear except for the essential role of Nkx2.2 in the specification of serotonergic neurons [8]. In fact individual Nkx2.2 and Nkx2.9 gene knock-out mice present rather mild phenotypes of motor neurons in the rhombencephalon suggesting redundant or overlapping functions [8, 10]. Here we provide genetic evidence that all branchiovisceral motor neurons in hindbrain are derived from Nkx2.2-expressing progenitor cells. We furthermore demonstrate that both Nkx2.2 and Nkx2.9 transcription factors contribute to the formation of branchial and visceral motor neurons with overlapping roles. Interestingly, a single intact Nkx2.2 gene copy in the absence of Nkx2.9 alleles appears sufficient to support formation of bvMNs in the murine hindbrain and ensures viability of adult animals [11]. Our observations indicate that Nkx2.2 and Nkx2.9 transcription factors are important to establish bvMNs in cranial hindbrain posterior to and including rhombomere 4. Both transcription factors appear dispensable for the identity of neuronal subtypes in the trigeminal nerve and presumably in the more rostral brain stem nerves, although both proteins are expressed along the entire rostro-caudal axis of the brain.

### Branchiovisceral motor neurons are derived from Nkx2.2^+^ p3 progenitor cells

Using Cre-mediated cell labeling we here formally demonstrate that motor neurons of the branchiovisceral subtype in the mammalian hindbrain are descendants of Nkx2.2 expressing progenitor cells. They are located within the p3 domain which is comprised of the most ventral neuronal progenitor cells, dorsally adjacent to the floor plate in the developing hindbrain (S3 Fig)[8]. The Nkx2.2^Cre^ mouse line carries a mutant Nkx2.2 allele in which the entire Nkx2.2 reading frame has been replaced by the Cre-recombinase (unpublished data). This Nkx2.2 allele appears to be reliably expressed in the correct neuronal progenitor cells, since broad co-expression of Nkx2.2 and Cre proteins is observed in the heterozygous Cre knock-in mouse (data not shown; Jarrar et al, in press, doi:10.1016/j.diff.2015.03.001). The Nkx2.2^Cre^ mutant mouse line was crossed to the appropriate Cre-responsive GFP reporter mouse to achieve genetic labeling of the Nkx2.2 cell lineage. We find postmitotic branchiovisceral motor neurons that co-express the cell lineage marker GFP together with the transcription factors Phox2b and Islet1 providing formal proof for the derivation of these cells from Nkx2.2^+^ progenitors (S3, S4 and S5 Figs)[27]. Moreover, these genetically marked cells exhibit all cellular characteristics of branchiovisceral motor neurons, namely the initial ventral location in the mantle zone [21], the dorsal migration [21, 28], projections of axons into the periphery via specific dorsolateral exit points [29], and clustering of cell somata in motor nuclei at distinct positions within the rhombomeric boundaries of the hindbrain [7]. Since all motor nuclei, which are composed of visceral and/or branchial motor neurons in caudal hindbrain are labeled without exception by the Nkx2.2-Cre mediated cell lineage marker, we conclude that all cranial motor neurons of the bvMN subtype are derived from Nkx2.2-expressing progenitor cells. Somatic motor neurons in cranial nerves are not labeled by the same cell lineage marker and therefore develop from different progenitor cells with no regional overlap. At present there are no molecular markers that would allow the distinction of branchial and visceral motor neurons. Our data provide good evidence that both subtypes are derived from the same cell lineage that initially expresses Nkx2.2 and resides in the p3 domain of all rhombomeres. For instance, motor nuclei, such as the dmnX that contains clusters of visceral motor neurons of the vagal nerve [20] and the nucleus ambiguus as well as the nucleus of the trigeminal nerve, both consisting exclusively of branchial motor neurons, are equally labeled by the cell lineage marker and are therefore derived from Nkx2.2^+^ progenitors (S4 and S5 Figs). Likewise, motor nuclei of the facial and the glossopharyngeal nerves, both containing mixed populations of branchial and visceral motor neurons, are identified by the Nkx2.2-Cre cell lineage marker (S5 Fig and data not shown).

In summary then this study clearly demonstrates that all branchiovisceral motor neurons between rhombomeres 7 and 2 in mouse hindbrain are derived from Nkx2.2^+^ neuronal progenitor cells within the p3 domain.

### Both Nkx2.2 and Nkx2.9 transcription factors contribute to the formation of branchiovisceral motor neurons

It has been suggested that the Nkx2.2 and Nkx2.9 transcription factors are key regulators for the determination of branchiovisceral cell fate [8, 10, 27]. Although ectopic expression of Nkx2.2 in caudal hindbrain leads to activation of the branchiovisceral differentiation program including the induction of Phox2b gene expression [27], Nkx2.2 knockout mice fail to show defects in the formation of branchiovisceral motor neurons [8]. Consequently all cranial nerves and motor nuclei of this subtype seem to develop fairly normal (Fig 1). Knockout mice for the homologous transcription factor Nkx2.9 present slight defects in the branchial spinal accessory nerve, which appears shorter and thinner than in wild type (Fig 1)[10]. The vagal nerve is also moderately affected in these mutant animals, as shown here and in a previous study [10]. Our present data on Nkx2.2; Nkx2.9 double-null embryos provide convincing evidence that both transcription factors contribute to the generation of branchiovisceral motor neurons in a redundant fashion, since most of these cells are missing in the double-null mutant. Moreover, our results suggest that both factors play an early role in specification of neuronal progenitor cells, as the entire p3 domain in E10.5 Nkx2.2^-/-^; Nkx2.9^-/-^ double-mutants contains Olig2^+^ sMNs that replace the bvMN progenitor cells. Both Nkx2.2 and Nkx2.9 factors operate also redundantly in the spinal cord where they specify floor plate and progenitor cells of glutamatergic V3 interneurons [11]. Both in spinal cord and in hindbrain it appears that the decision which of the individual neuronal programs is executed in the p3 domain involves repression of Olig2 expression by Nkx2.2 and/or Nkx2.9 thereby preventing cell fate of somatic motor neurons [25, 30]. Whether or not the transcription factors Nkx2.2 and Nkx2.9 are in addition capable and sufficient to activate the program of bvMN specification has not been addressed here.

Our cell lineage analysis demonstrates a switch in cell fate in the absence of Nkx2.2 and Nkx2.9 arguing that the expansion of sMNs and the reduction of bvMNs is unlikely results from increased cell proliferation and apoptosis of the respective neuronal subpopulations. Similar to findings in the spinal cord [11] we did not observe an augmented rate of apoptosis among motor neurons and their precursors in absence of the Nkx2 transcription factors (data not shown). Moreover, somatic motor neurons derived from the p3 domain in double-mutants behave identical to the intrinsic sMNs, as their axons project through ventral exit points and follow the routes of the normal somatic motor neuron nerves into the periphery.

### The requirement of Nkx2.2 and Nkx2.9 transcription factors for cell lineage determination of branchiovisceral motor neurons changes along the posterior-anterior axis of the hindbrain

It has been shown previously that the formation of V3 interneurons in spinal cord is dependent on Nkx2.2/Nkx2.9-expressing progenitor cells in the p3 domain [11]. Here we demonstrate that cells in the p3 domain of hindbrain give rise to branchiovisceral motor neurons that subsequently form cranial nerves. In the absence of the transcription factors Nkx2.2 and Nkx2.9 neurons of purely somatic motor nerves, such as the hypoglossal (XII) and motor nerves in the spinal cord (C1-C4) are born ectopically in the p3 domain and therefore carry the Nkx2.2-Cre mediated cell lineage marker (Fig 8). As expected lineage-marked bvMNs are also detected in the more caudal dmnx and the nucleus ambiguus as well as in the more rostral motor nuclei of the facial and trigeminal nerves indicating that they all are derived from Nkx2.2 expressing progenitor cells (S4 and S5 Figs). We have not examined in detail the more anterior nerves of the brain stem but assume that they also originate from the most ventral population (p3) of neuronal progenitor cells, except for the neurons of the oculomotor complex which contains somatic motor neurons. It is absolutely clear that the transcription factors Nkx2.2 and Nkx2.9 are required to specify branchial and visceral motor neurons in caudal hindbrain, since mouse embryos deficient for both genes essentially lack the dmnX and the nucleus ambiguus. In contrast motor nuclei of the facial and the trigeminal nerves are only partly or not at all affected in double-null mutants, respectively (Fig 4 and S4 and S5 Figs). Our observations are compatible with the idea that the strict requirement for both Nkx2 factors in determining subtypes of neuronal progenitor cells applies only to the caudal hindbrain excluding the trigeminal nucleus and presumably all nuclei rostral to it. It is tempting to speculate that these rostral nerves in the brainstem depend on determination mechanisms other than those involving Nkx2.2 or Nkx2.9 proteins, even though both factors are expressed in the corresponding progenitor cells. Presently it is unclear which control proteins ascertain that branchial and visceral motor neurons are generated rostral to rhombomere 4. The facial motor nucleus seems to be located in a zone of mechanistic transition, since some residual branchial motor neurons are still present there, even when Nkx2.2 and Nkx2.9 proteins are entirely ablated.

## Supporting Information

S1 Fig Nkx2.2^-/-^; Nkx2.9^-/-^ double-deficient embryos lack distinct cranial nerves. 2H3 antibody staining of neurofilament on whole-mounts of wild type (A) and mutant (B) embryos. Note the loss of vagal (X) and spinal accessory (XI) nerves. The trigeminal (V), facial/vestibulocochlear (VII/VIII), glossopharyngeal (IX), and hypoglossal (XII) nerves appear not affected. The first spinal nerve in the cervical region is called C1. Scale bar: 400 μm.
(TIF)

S2 Fig Significant reduction of Phox2b expressing bvMN progenitor cells at the ventral midline of caudal hindbrain in Nkx2.2; Nkx2.9 double-deficient mouse embryos. Open-book preparations of hindbrain (anterior at top, posterior at bottom) from E10.5 embryos were subjected to immunohistochemistry using the Phox2b-specific antibody. Phox2b expression in the ventral p3 domain (center of images) is significantly reduced within and caudal to rhombomere 4 of Nkx2.2; Nkx2.9 double-deficient (E) embryos compared to wild type (A), single (B, C) and heterozygous-homozygous compound mutants (D). Arrow in A and solid arrowhead in E mark the position of rhombomere 7. Rhombomere 4 is also indicated (A, E). Note, that laterally (*) as well as dorsally (open arrowheads in A and E) located Phox2b-expressing cells that do not represent bvMNs appear unaltered in mutant hindbrains. Scale bar: 400 μm.
(TIF)

S3 Fig Isl1^+^ and Phox2b^+^ positive bvMNs in hindbrain originate from Nkx2.2-expressing progenitor cells in the p3 domain. Genetic cell lineage analysis on a transversal section (rhombomere 7) of a hemizygous Nkx2.2-Cre knock-in control mouse demonstrates membrane-associated GFP expression in neuronal progenitor cells of the ventricular zone and in differentiated motor neurons of the mantle zone. Note that mature neurons co-express Isl1 (red) and Phox2b (blue) indicating that they belong to the branchial or/or visceral subtype of motor neurons. Some of these cells have initiated the dorsal migration toward the final location in the motor nuclei of cranial nerves.
(TIF)

S4 Fig The branchial motor nucleus of the trigeminal nerve is derived from bvMN progenitor cells but does not depend on Nkx2.2 and Nkx2.9 to maintain the correct motor neuron subtype. Serial sections of hindbrain from a Nkx2.2; Nkx2.9 double-deficient E12.5 mouse embryo were triple stained with fluorescent antibodies to the cell lineage marker membrane-bound GFP (green), the motor neuron marker Islet1 (red), and the bvMN-specific transcription factor Phox2b (blue). Note that all motor neurons in the double-mutant mouse remain positive for the bvMN marker Phox2b and fail to express the sMN marker Hb9. Scale bar: 50 μm.
(TIF)

S5 Fig A subset of bvMNs in the motor nucleus of the facial nerve develops in the absence of Nkx2.2 and Nkx2.9 transcription factors. Sections of the facial nucleus from E12.5 control (A, B) and Nkx2.2; Nkx2.9 double-knockout (C, D) embryos were triple stained using fluorescent antibodies directed against GFP (green), Islet1 (red), and Phox2b (blue). Note that residual bvMN neurons remain present in the facial nucleus even when both Nkx2.2 and Nkx2.9 proteins have been ablated genetically. The dotted lines mark the pial boundaries. Scale bar: 50 μm.
(TIF)
