# Supplementary figures and images for: Nkx2.2 and Nkx2.9 Are the Key Regulators to Determine Cell Fate of Branchial and Visceral Motor Neurons in Caudal Hindbrain
Source: PLoS One. 2015 Apr 28;10(4):e0124408. doi: 10.1371/journal.pone.0124408 (PMC4412715; doi:10.1371/journal.pone.0124408)

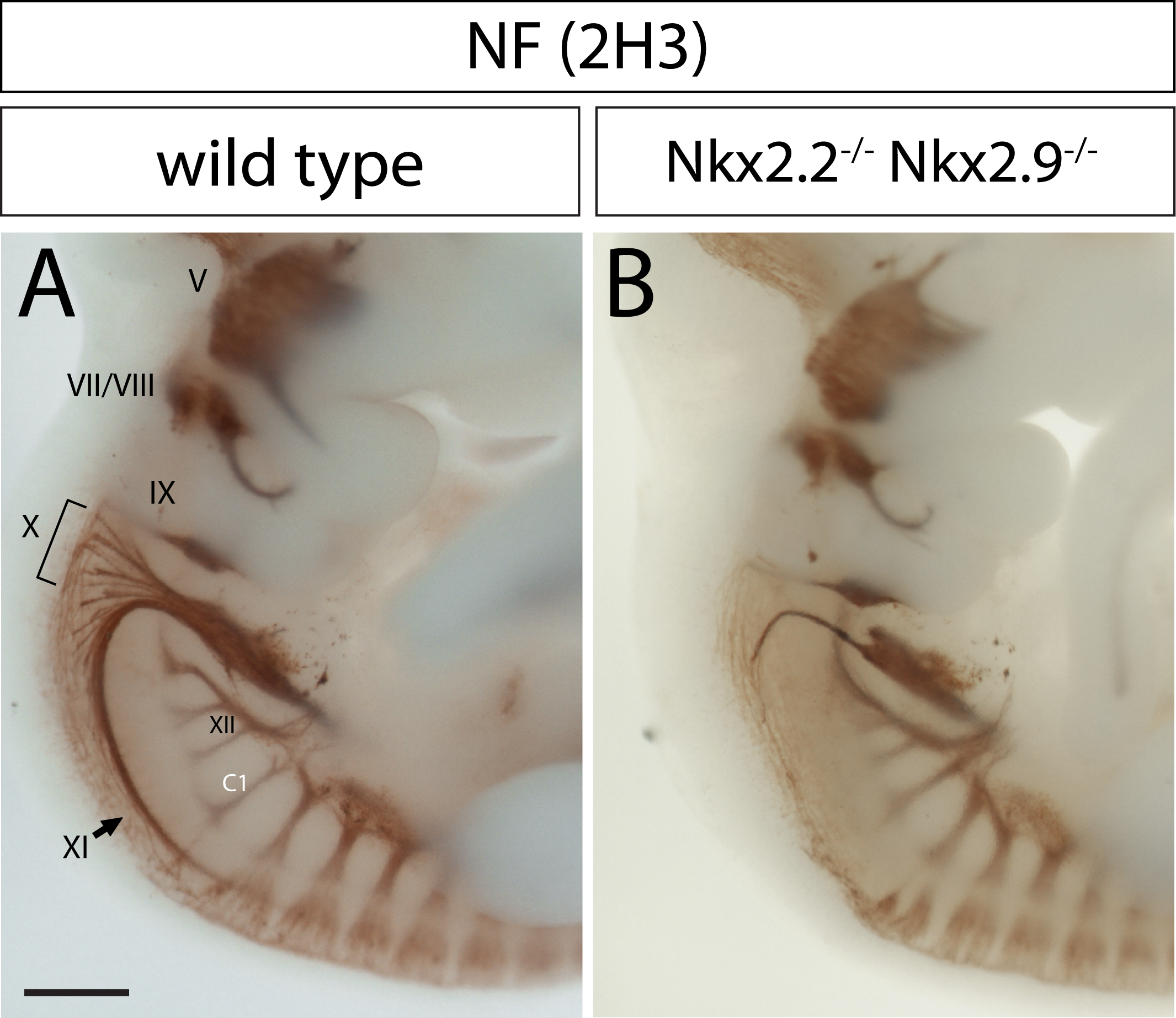

Supplement: S1 Fig — 2H3 antibody staining of neurofilament on whole-mounts of wild type (A) and mutant (B) embryos. Note the loss of vagal (X) and spinal accessory (XI) nerves. The trigeminal (V), facial/vestibulocochlear (VII/VIII), glossopharyngeal (IX), and hypoglossal (XII) nerves appear not affected. The first spinal nerve in the cervical region is called C1. Scale bar: 400 μm. (TIF) [file pone.0124408.s001.tif]

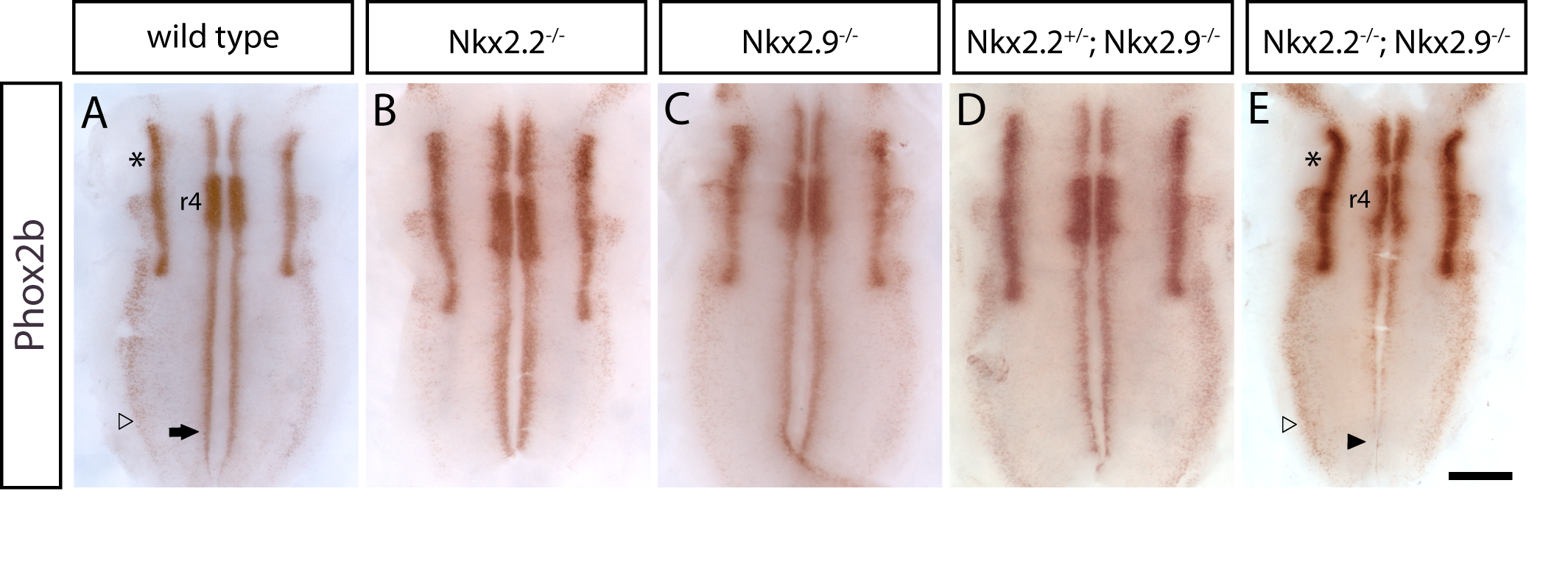

Supplement: S2 Fig — Open-book preparations of hindbrain (anterior at top, posterior at bottom) from E10.5 embryos were subjected to immunohistochemistry using the Phox2b-specific antibody. Phox2b expression in the ventral p3 domain (center of images) is significantly reduced within and caudal to rhombomere 4 of Nkx2.2; Nkx2.9 double-deficient (E) embryos compared to wild type (A), single (B, C) and heterozygous-homozygous compound mutants (D). Arrow in A and solid arrowhead in E mark the position of rhombomere 7. Rhombomere 4 is also indicated (A, E). Note, that laterally (*) as well as dorsally (open arrowheads in A and E) located Phox2b-expressing cells that do not represent bvMNs appear unaltered in mutant hindbrains. Scale bar: 400 μm. (TIF) [file pone.0124408.s002.tif]

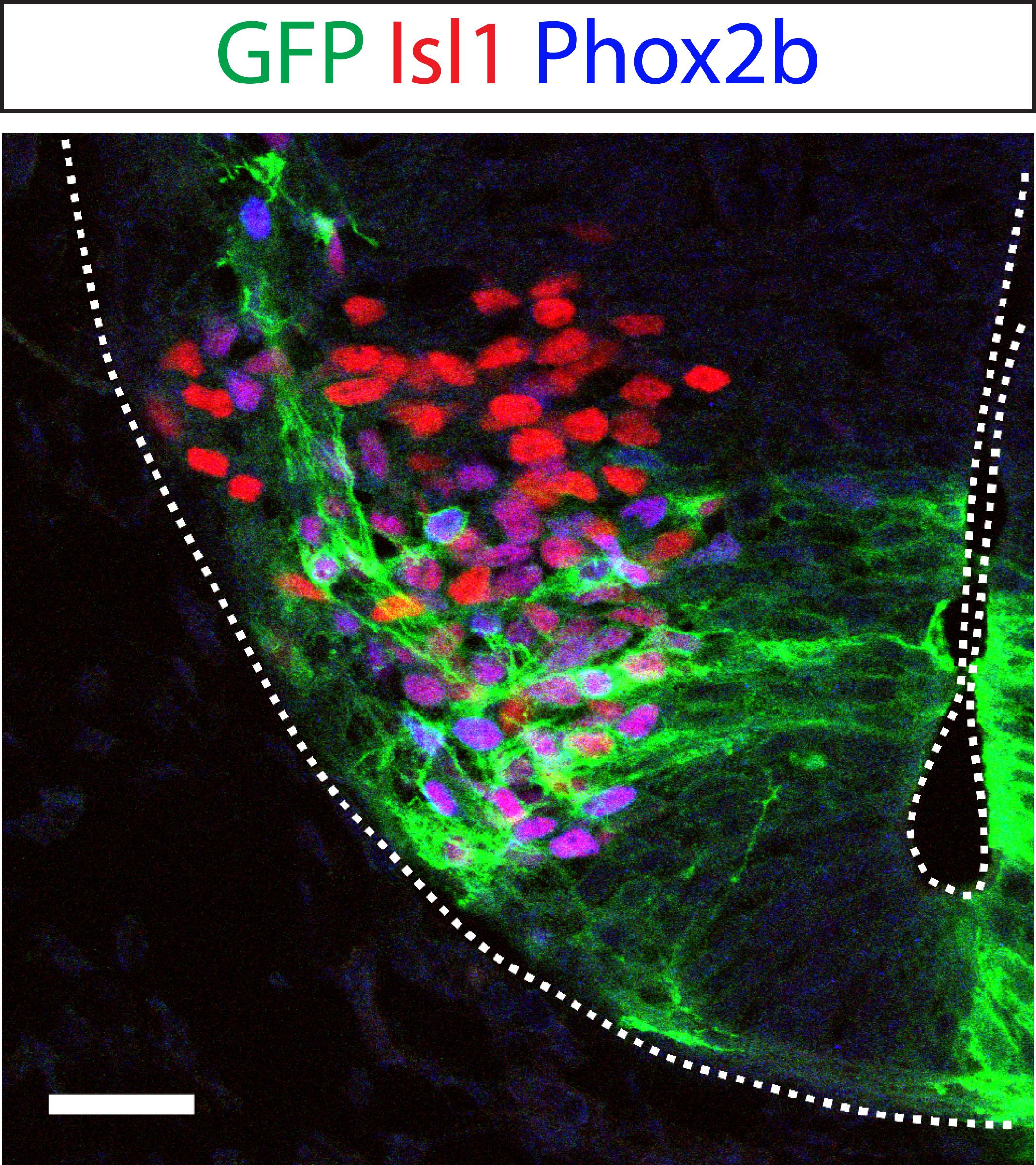

Supplement: S3 Fig — Genetic cell lineage analysis on a transversal section (rhombomere 7) of a hemizygous Nkx2.2-Cre knock-in control mouse demonstrates membrane-associated GFP expression in neuronal progenitor cells of the ventricular zone and in differentiated motor neurons of the mantle zone. Note that mature neurons co-express Isl1 (red) and Phox2b (blue) indicating that they belong to the branchial or/or visceral subtype of motor neurons. Some of these cells have initiated the dorsal migration toward the final location in the motor nuclei of cranial nerves. (TIF) [file pone.0124408.s003.tif]

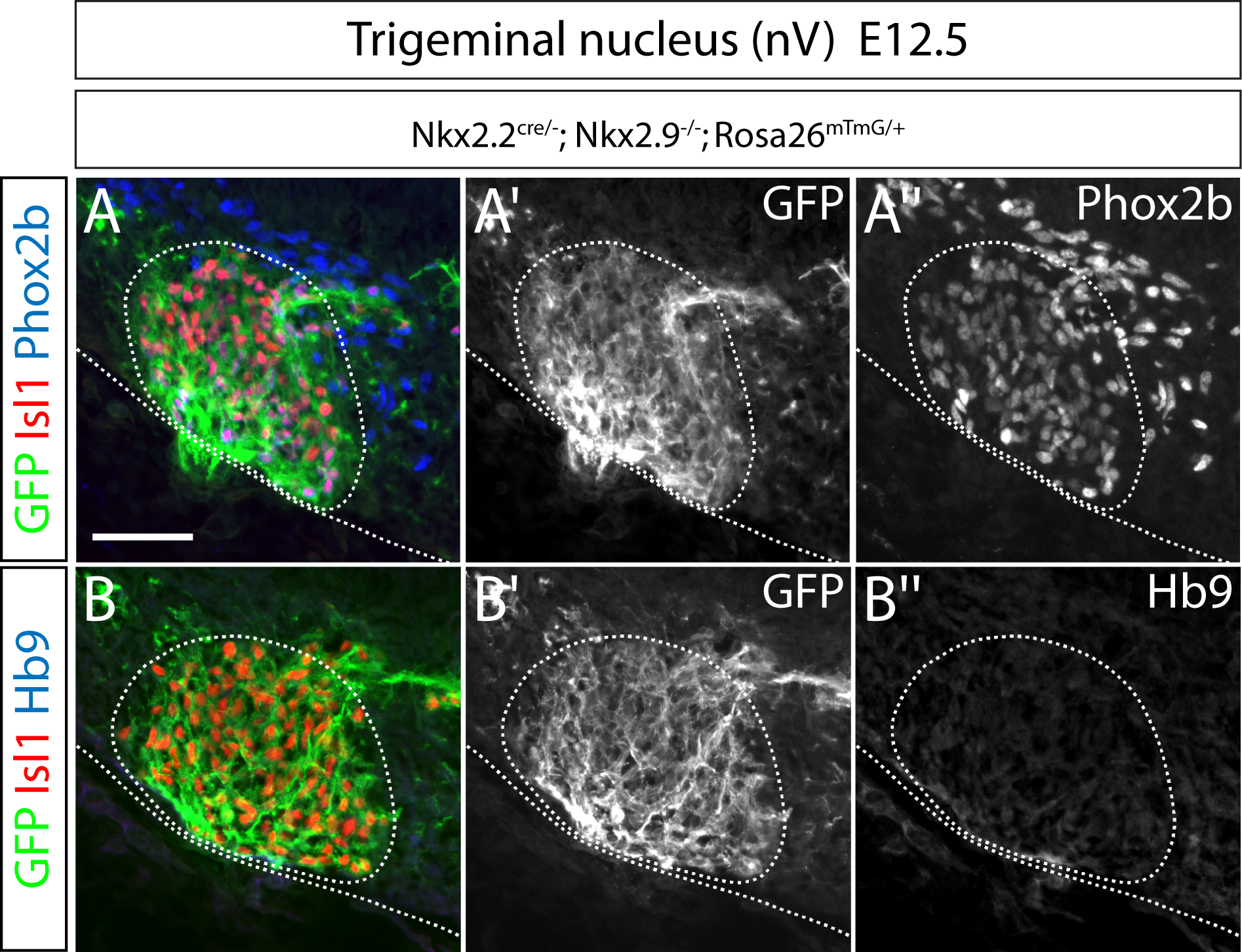

Supplement: S4 Fig — Serial sections of hindbrain from a Nkx2.2; Nkx2.9 double-deficient E12.5 mouse embryo were triple stained with fluorescent antibodies to the cell lineage marker membrane-bound GFP (green), the motor neuron marker Islet1 (red), and the bvMN-specific transcription factor Phox2b (blue). Note that all motor neurons in the double-mutant mouse remain positive for the bvMN marker Phox2b and fail to express the sMN marker Hb9. Scale bar: 50 μm. (TIF) [file pone.0124408.s004.tif]

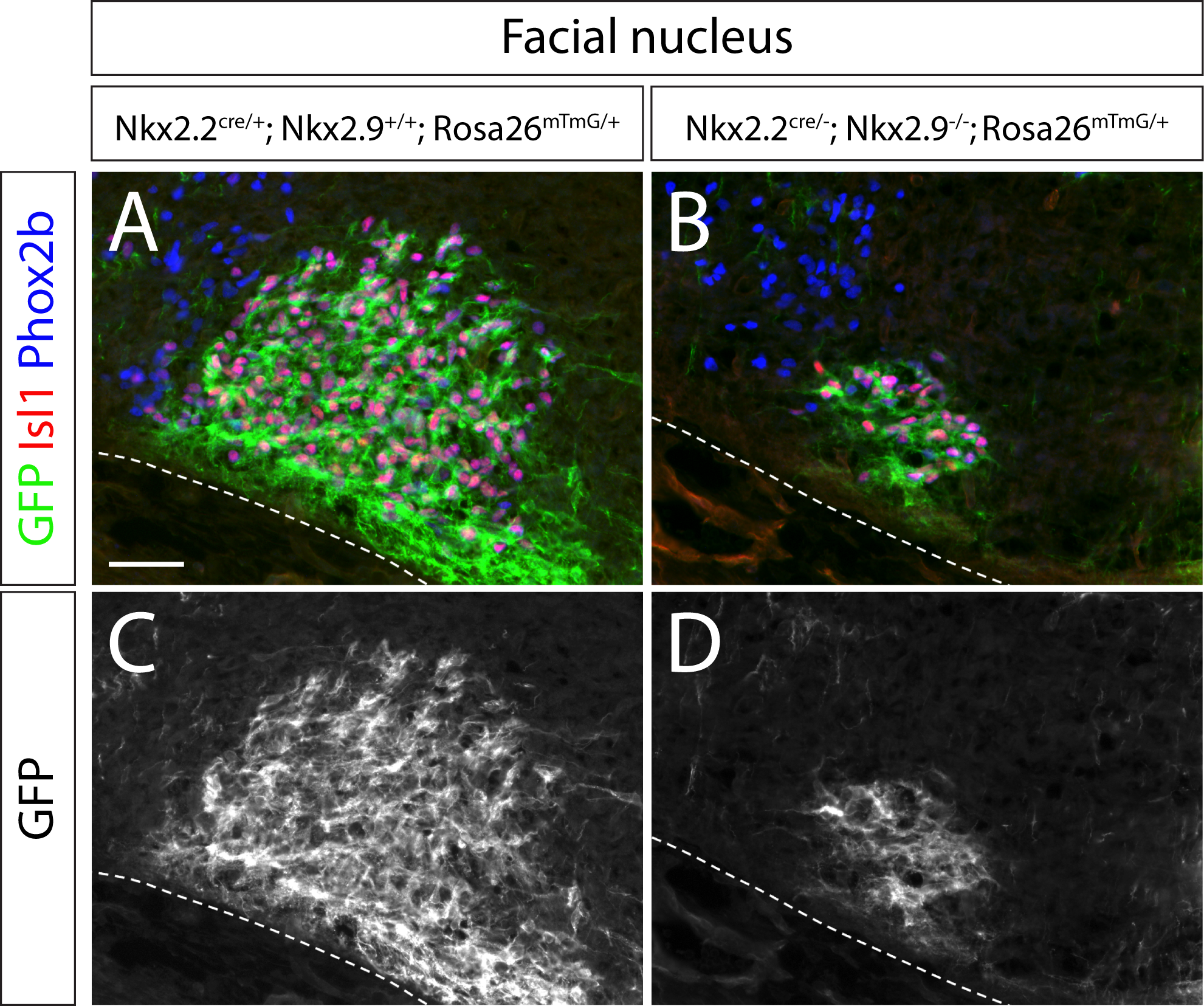

Supplement: S5 Fig — Sections of the facial nucleus from E12.5 control (A, B) and Nkx2.2; Nkx2.9 double-knockout (C, D) embryos were triple stained using fluorescent antibodies directed against GFP (green), Islet1 (red), and Phox2b (blue). Note that residual bvMN neurons remain present in the facial nucleus even when both Nkx2.2 and Nkx2.9 proteins have been ablated genetically. The dotted lines mark the pial boundaries. Scale bar: 50 μm. (TIF) [file pone.0124408.s005.tif]
